# Supplementary material for: Exploring the Genetic Causality of Discordant Phenotypes in Familial Apparently Balanced Translocation Cases Using Whole Exome Sequencing
Source: Genes (Basel). 2022 Dec 27;14(1):82. doi: 10.3390/genes14010082 (PMC9859009; doi:10.3390/genes14010082)
Supplement: Supplementary file 1 [file genes-14-00082-s001.zip › Document S1.pdf]

Document S1

In-house Bioinformatic Exome Analysis Pipeline

An in-house pipeline used in this study was mainly based on the Best Practices provided by the Genome Analysis Toolkit (GATK), v3.3 (https://software.broadinstitute.org/gatk/) [1]. Once the single nucleotide polymorphisms (SNPs) and small insertions or deletions (indels) were identified, the variant effect prediction (VEP) tool [2] was applied and further annotation and interpretation and filtering was handled by GEMINI - version 0.20.1 [3]. The pipeline described here (Figure S1) was based on the available resources and tools and runs on Cytera HPC clusters of the Cyprus Institute* at the time of analysis. Cytera was an IPM Hybrid CPU/GPU cluster with 98 twelve core compute nodes and 18 dual-GPU nodes and is provided as part of preparatory access project (https://castorc.cyi.ac.cy/infrastructure#HPCSystems).

WES Data Quality Check, Pre-processing, and Alignment

The freely-available FastQC tool (v0.10.1) was used to perform some basic quality control checks on the raw sequencing reads (FASTQ files) produced from WES (https://www.bioinformatics.babraham.ac.uk/projects/fastqc/). Low-quality base calls and adapter sequences were trimmed in the FASTQ files using Cutadapt (v1.5) [4]. The above-mentioned tools were used within a single wrapper tool the Trim Galore tool (https://www.bioinformatics.babraham.ac.uk/projects/trim_galore/) in a single command. Burrows Wheeler Aligner (BWA)-MEM with default parameters (http://bio-bwa.sourceforge.net/) was used to align high-quality paired-end reads to the human reference genome hg19 [5]. The Sequencing Alignment Map (SAM) files produced by the BWA-MEM tool were then converted to the binary format BAM files using SAMTools (v0.1.18) [6]. Next, the Picard MarkDuplicates tool (v1.109) (Broad Institute) was used to mark optical and PCR duplicates (http://broadinstitute.github.io/picard/), and thus, avoid any biases in subsequent variant calling. Base quality score recalibration was then performed by using the GATK BaseRecalibrator tool (Broad Institute) in order to account for systematic technical errors affecting base quality scores, and thus, improve variant call accuracy.

* “This work was supported by the Cy-Tera Project (ΝΕΑ ΥΠΟΔΟΜΗ/ΣΤΡΑΤΗ/0308/31), which is co-funded by the European Regional Development Fund and the Republic of Cyprus through the Research Promotion Foundation.”

Variant Calling and Annotation

Variant calling for single nucleotide variants (SNVs) and small insertion-deletions (indels) was performed using the GATK HaplotypeCaller in ERC mode in each sample separately. The output is in genomic VCF format (gVCF) containing extra information that enhances the variant analysis. Samples of the same run are used as input in the GATK GenotypeGVCFs tool that performs the multi-sample joint aggregation step and merges the records together in a sophisticated manner in order to produce correct genotype likelihoods, re-genotype the newly merged record, and then re-annotation in vcf format. GATK Hardfiltering is then performed separately on SNPs and INDELs (GATK SelectVariants) using the parameters recommended by GATK for exome sequencing analysis: Parameters for SNPs: "QualByDepth (QD) < 2.0 || FisherStrand (FS) > 60.0 || RMSMappingQuality (MQ) < 40.0 || MappingQualityRankSumTest (MQRankSum) < -12.5 || ReadPosRankSumTest (ReadPosRankSum) < -8.0 || HaplotypeScore >13.0"; Parameters for Indels: "QD < 2.0 || FS > 200.0 || ReadPosRankSum < -20.0". Filtered SNPs and indels were then combined into a single Variant Call Format (VCF) file using the GATK CombineVariants tool.

For variant annotation GEMINI version 0.20.1 was used, a flexible framework for exploring variation [3]. Prior loading to GEMINI, the vcf file is annotated using VEP (Ensembl release 81) [2]. The library created can be then searched and filtered using SQL queries and results can be retrieved and visualized in excel format. Potential candidate variants from the vcf are inspected along with the aligned reads within the bam files using the Integrative Genomics Viewer (IGV) tool (http://software.broadinstitute.org/software/igv/) and variants are compared with the variants found in local population to exclude local polymorphisms.

Filtering and further processing

In-house filtering were implemented and prioritised using inheritance mode, several *in silico* prediction tools, such as CADD (https://cadd.gs.washington.edu/), PolyPhen (version 2.2.2) (http://genetics.bwh.harvard.edu/pph2/) and SIFT (version 5.2.2) (https://sift.bii.a-star.edu.sg/). For population frequency, filtering the gnomAD exomes r2.0.1 (https://gnomad.broadinstitute.org/) and 1000 Genomes (Phase 3) (http://phase3browser.1000genomes.org/index.html) were used, as well as an in–house database. OMIM (https://omim.org/) and ClinVar (https://www.ncbi.nlm.nih.gov/clinvar/) aided in the identification of known pathogenic variants or established genes contributing to known clinical phenotypes.

Peddy tool (v 0.4.2) was used after each run to derive an accurate prediction of the individual's sex and to check relatedness between samples from the same family/trio [7]. The latter feature is essential when discovering *de novo* variants.

Additional tools were used to assess the impact of disease-candidate variants such as MutationTaster (http://www.mutationtaster.org) [8] and Human Splicing Finder (http://www.umd.be/HSF3) [9].

In-silico Panels for Whole Exome Sequencing Data Filtering

Depending on the presented phenotypes in each ABT family analysed, *in silico* panels were created for WES data filtering purposes. In ABT families 1, 2, and 3, a list of 729 intellectual disability-related genes were used, collectively extracted from genes included in the Greenwood Genetic Center X-linked intellectual disability panel (n=114) (https://www.ggc.org/) (Supplementary Table S1) and genes linked to isolated ID and ID-associated disorders published by Vissers et al., 2015 (n=709) [10]. In ABT family 4, a list of 40 genes known to cause polysyndactyly/synpolydactyly, orofaciodigital syndrome and Joubert syndrome were used (Supplementary Table S2).

CNV detection from WES data

CNV detection was performed using ExomeDepth (v. 1.1.15) [11]. For this analysis an in-house R script was implemented using R version 3.6. The analysis was performed with default parameters using bam files of the run. For each reference set, 5-10 samples were used depending on the available unrelated samples in the batch run. For potential CNV calls on X chromosome only samples from the same sex were correlated. Results were filtered using Bayes factor (BF) >50 and each call was further annotated using the online VEP tool (Ensembl release 81) and BioMart (http://grch37.ensembl.org/biomart/martview/).

Supplementary Figures

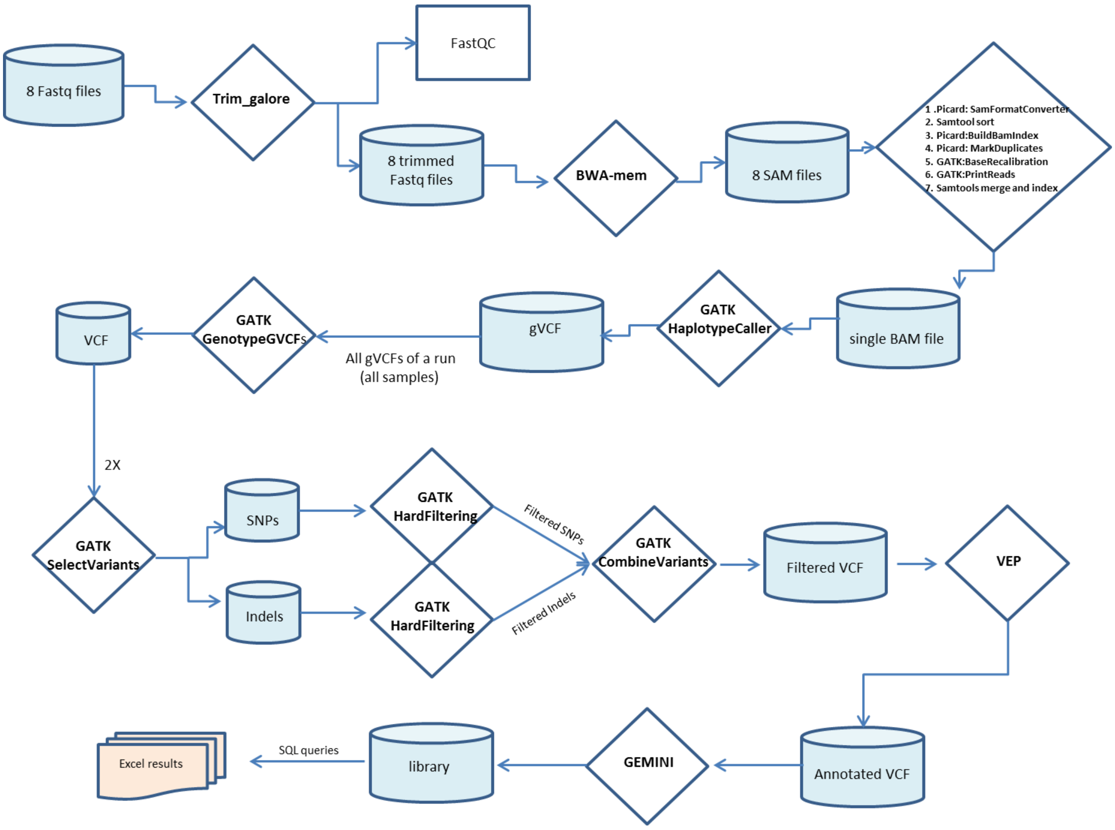

Figure S1. In-house bioinformatics pipeline for variant calling of an exome sequencing paired-end experiment using the Illumina NextSeq500 platform.

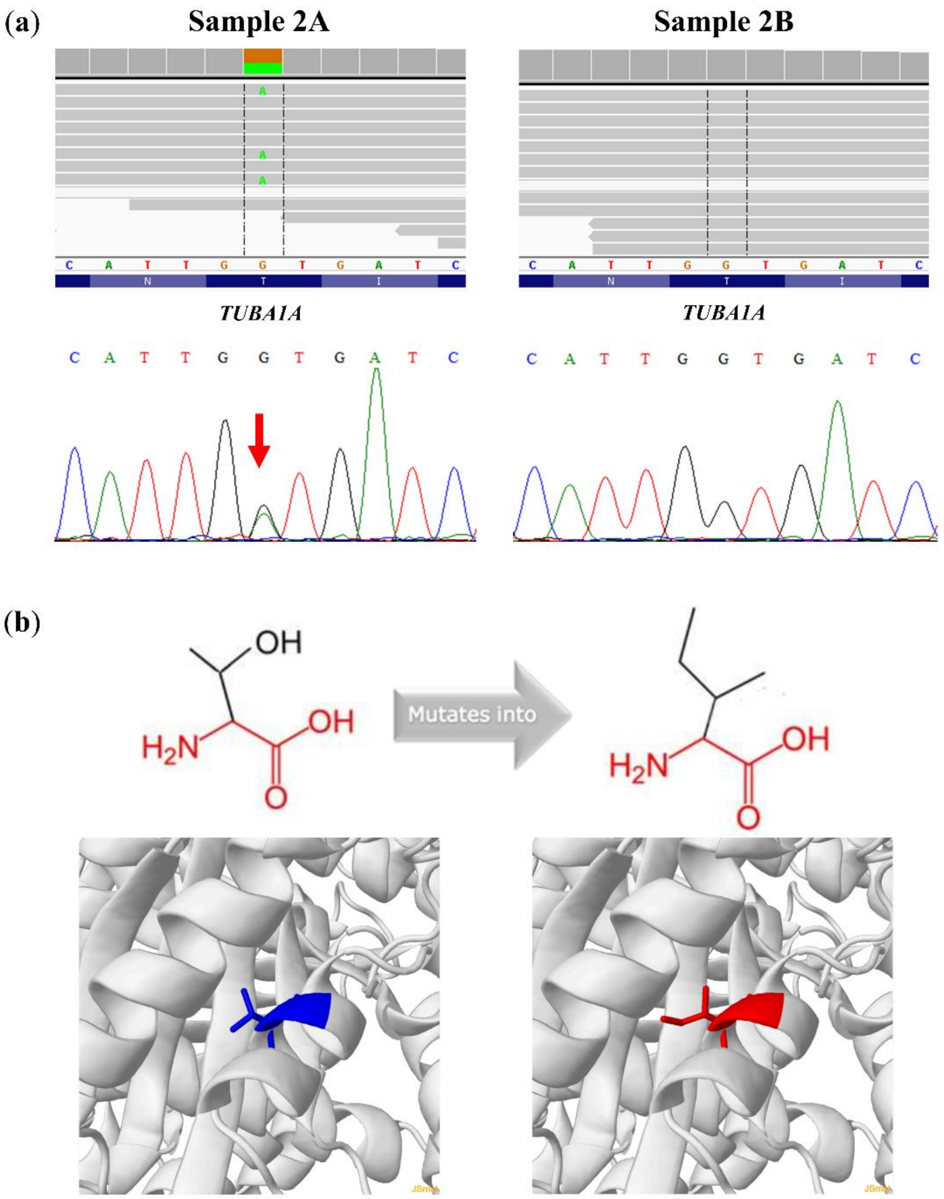

Figure S2: Family 2 candidate variant investigation. (a) Top panel: IGV screenshot depicting the patient-specific *TUBA1A* missense variant identified by WES. Lower panel: Sanger sequencing confirmed that the affected sibling (sample 2A) was heterozygous for the mutated allele (red arrow), while the non-affected sibling (sample 2B) was homozygous for the reference allele; (b) Top panel: Schematic structures of the wild-type threonine (left) and the mutant isoleucine (right) amino acids. The backbone, which is the same for each amino acid, is coloured red. The side chain, unique for each amino acid, is coloured black. Lower panel: Structural analysis of the NP_006000.2:p.(Thr292Ile) variant (Protein Data Bank code: 5JCO); the mutant isoleucine (right) affects hydrogen bonds formed by the wild-type threonine (blue), thus disturbing correct protein folding.

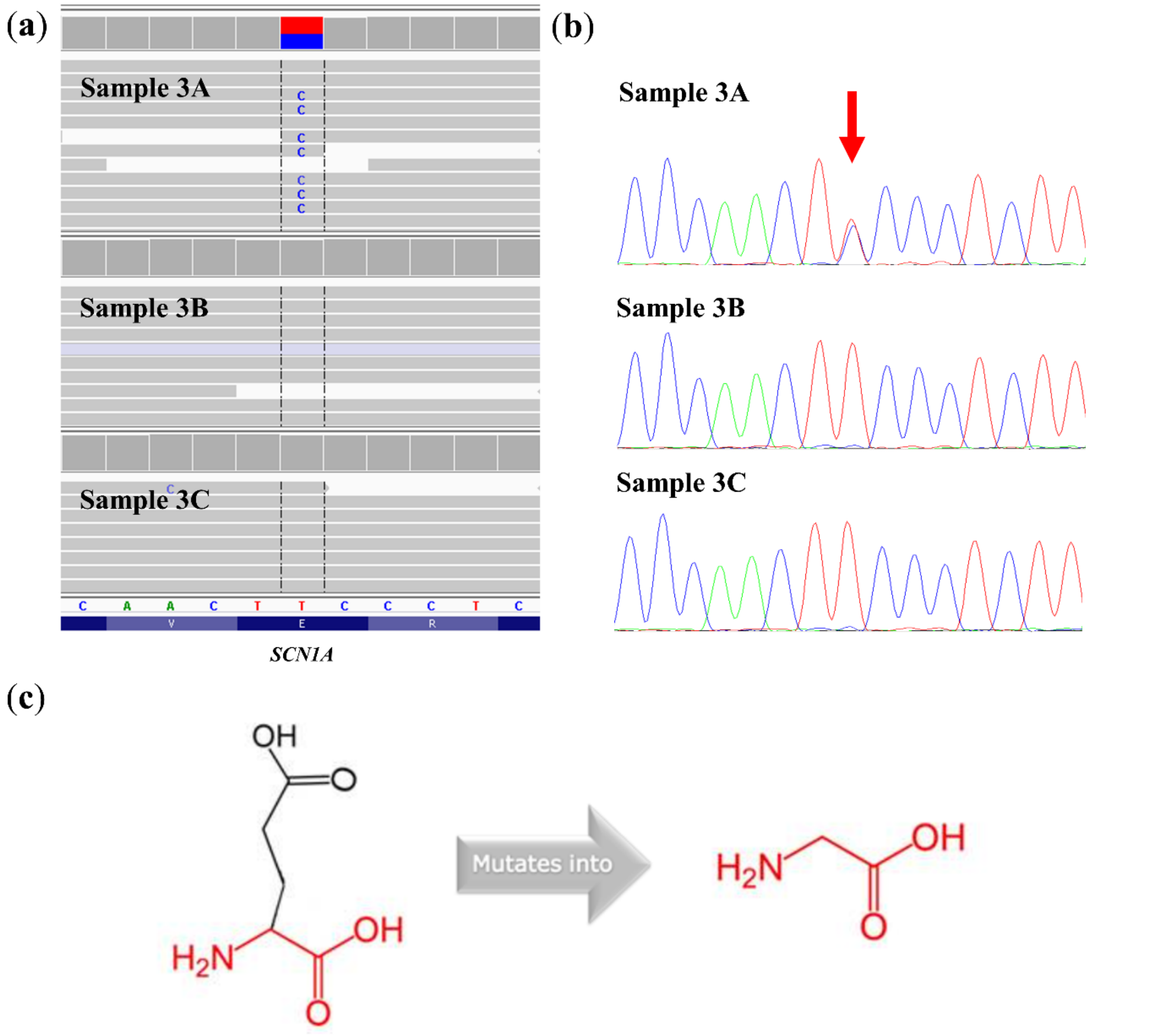

Figure S3: Family 3 candidate variant investigation. (a) IGV screenshot depicting the patient-specific *SCN1A* candidate variant identified by WES; (b) Sanger sequencing confirmed that the proband of family 3 (sample 3A) is heterozygous for the mutated allele (red arrow), while the non-affected mother (sample 3B) and male sibling (sample 3C) are both homozygous for the reference allele; (c) Schematic structures of the wild-type glutamic acid (left) and the mutant glycine (right) amino acids. The backbone, which is the same for each amino acid, is coloured red. The side chain, unique for each amino acid, is coloured black.

References

1. McKenna, A.; Hanna, M.; Banks, E.; Sivachenko, A.; Cibulskis, K.; Kernytsky, A.; Garimella, K.; Altshuler, D.; Gabriel, S.; Daly, M.; et al. The Genome Analysis Toolkit : A MapReduce framework for analyzing next-generation DNA sequencing data. *Genome Res.* **2010**, *20*, 1297-1303, doi:10.1101/gr.107524.110.20.
2. McLaren, W.; Gil, L.; Hunt, S.E.; Riat, H.S.; Ritchie, G.R.S.; Thormann, A.; Flicek, P.; Cunningham, F. The Ensembl Variant Effect Predictor. *Genome Biol.* **2016**, *17*, 122, doi:10.1186/s13059-016-0974-4.
3. Paila, U.; Chapman, B.A.; Kirchner, R.; Quinlan, A.R. GEMINI: Integrative Exploration of Genetic Variation and Genome Annotations. *PLoS Comput. Biol.* **2013**, *9*, e1003153, doi:10.1371/journal.pcbi.1003153.
4. Martin, M. Cutadapt removes adapter sequences from high-throughput sequencing reads. *2011* **2011**, *17*, 3, doi:10.14806/ej.17.1.200.
5. Li, H.; Durbin, R. Fast and accurate short read alignment with Burrows-Wheeler transform. *Bioinformatics* **2009**, *25*, 1754-1760, doi:10.1093/bioinformatics/btp324.
6. Li, H.; Handsaker, B.; Wysoker, A.; Fennell, T.; Ruan, J.; Homer, N.; Marth, G.; Abecasis, G.; Durbin, R.; Genome Project Data Processing Subgroup, G.P.D.P. The Sequence Alignment/Map format and SAMtools. *Bioinformatics* **2009**, *25*, 2078-2079, doi:10.1093/bioinformatics/btp352.
7. Pedersen, B.S.; Quinlan, A.R. Who's Who? Detecting and Resolving Sample Anomalies in Human DNA Sequencing Studies with Peddy. *Am. J. Hum. Genet.* **2017**, *100*, 406-413, doi:10.1016/J.AJHG.2017.01.017.
8. Schwarz, J.M.; Cooper, D.N.; Schuelke, M.; Seelow, D. MutationTaster2: mutation prediction for the deep-sequencing age. *Nat. Methods* **2014**, *11*, 361-362, doi:10.1038/nmeth.2890.
9. Desmet, F.O.; Hamroun, D.; Lalande, M.; Collod-Bëroud, G.; Claustres, M.; Béroud, C. Human Splicing Finder: An online bioinformatics tool to predict splicing signals. *Nucleic Acids Res.* **2009**, *37*, 1-14, doi:10.1093/nar/gkp215.
10. Vissers, L.E.L.M.; Gilissen, C.; Veltman, J.A. Genetic studies in intellectual disability and related disorders. *Nat. Rev. Genet.* **2015**, *17*, 9-18, doi:10.1038/nrg3999.
11. Plagnol, V.; Curtis, J.; Epstein, M.; Mok, K.Y.; Stebbings, E.; Grigoriadou, S.; Wood, N.W.; Hambleton, S.; Burns, S.O.; Thrasher, A.J.; et al. A robust model for read count data in exome sequencing experiments and implications for copy number variant calling. *Bioinformatics* **2012**, *28*, 2747-2754, doi:10.1093/BIOINFORMATICS/BTS526.
