# Supplementary material for: Exploring the Genetic Causality of Discordant Phenotypes in Familial Apparently Balanced Translocation Cases Using Whole Exome Sequencing
Source: Genes (Basel). 2022 Dec 27;14(1):82. doi: 10.3390/genes14010082 (PMC9859009; doi:10.3390/genes14010082)
Supplement: Supplementary file 1 [file genes-14-00082-s001.zip › genes-2026721-FC done.pdf]

genes

Article

Exploring the Genetic Causality of Discordant Phenotypes in Familial Apparently Balanced Translocation Cases Using Whole Exome Sequencing

Constantia Aristidou [1], Athina Theodosiou [1], Angelos Alexandrou [1], Ioannis Papaevripidou [1], Paola Evangelidou [1], Zoe Kosmaidou-Aravidou [2], Farkhondeh Behjati [3], Violetta Christophidou-Anastasiadou [4,5], George A. Tanteles [5] and Carolina Sismani [1,*]

[1] Department of Cytogenetics and Genomics, The Cyprus Institute of Neurology and Genetics, 2371 Nicosia, Cyprus
[2] Department of Genetics, Alexandra Hospital, 11528 Athens, Greece
[3] Genetics Research Center, University of Social Welfare and Rehabilitation Sciences, Tehran 1985713871, Iran
[4] Department of Clinical Genetics, Archbishop Makarios III Medical Centre, 2012 Nicosia, Cyprus
[5] Department of Clinical Genetics and Genomics, The Cyprus Institute of Neurology and Genetics, 2371 Nicosia, Cyprus
* Correspondence: csismani@cing.ac.cy

Abstract: Familial apparently balanced translocations (ABTs) are usually not associated with a phenotype; however, rarely, ABTs segregate with discordant phenotypes in family members carrying identical rearrangements. The current study was a follow-up investigation of four familial ABTs, where whole exome sequencing (WES) was implemented as a diagnostic tool to identify the underlying genetic aetiology of the patients' phenotypes. Data were analysed using an in-house bioinformatics pipeline alongside VarSome Clinical. WES findings were validated with Sanger sequencing, while the impact of splicing and missense variants was assessed by reverse-transcription PCR and in silico tools, respectively. Novel candidate variants were identified in three families. In family 1, it was shown that the de novo pathogenic *STXBP1* variant (NM_003165.6:c.1110+2T>G) affected splicing and segregated with the patient's phenotype. In family 2, a likely pathogenic *TUBA1A* variant (NM_006009.4:c.875C>T, NP_006000.2:p.(Thr292Ile)) could explain the patient's symptoms. In family 3, an *SCN1A* variant of uncertain significance (NM_006920.6:c.5060A>G, NP_008851.3:p.(Glu1687Gly)) required additional evidence to sufficiently support causality. This first report of WES application in familial ABT carriers with discordant phenotypes supported our previous findings describing such rearrangements as coincidental. Thus, WES can be recommended as a complementary test to find the monogenic cause of aberrant phenotypes in familial ABT carriers.

Keywords: familial apparently balanced translocations; whole exome sequencing; RT-PCR; *STXBP1*; *TUBA1A*; *SCN1A*

Citation: Aristidou, C.; Theodosiou, A.; Alexandrou, A.; Papaevripidou, I.; Evangelidou, P.; Kosmaidou-Aravidou, Z.; Behjati, F.; Christophidou-Anastasiadou, V.; Tanteles, G.A.; Sismani, C. Exploring the Genetic Causality of Discordant Phenotypes in Familial Apparently Balanced Translocation Cases Using Whole Exome Sequencing. *Genes* **2023**, *14*, 82. https://doi.org/10.3390/genes14010082

Academic Editor: Yuval Itan

Received: 27 October 2022
Revised: 23 December 2022
Accepted: 24 December 2022
Published: 27 December 2022

Copyright: © 2022 by the author. Licensee MDPI, Basel, Switzerland. This article is an open access article distributed under the terms and conditions of the Creative Commons Attribution (CC BY) license (https://creativecommons.org/licenses/by/4.0/).

1. Introduction

The great majority of apparently balanced translocation (ABT) carriers are phenotypically normal; however, they are at risk of experiencing infertility, recurrent miscarriages, and stillbirths, or having affected offspring due to meiotic malsegregation of the normal and derivative chromosomes. Subsequently, this leads to the generation of unbalanced gametes and zygotes with partial aneuploidy of the translocated chromosomes [1,2]. In the case of prenatally detected de novo ABTs, an associated phenotypic risk of ~6.7% was initially estimated [3]; this was later revised to a morbidity

risk of 27%, according to a long-term follow-up study [4]. In contrast, in couples/families carrying simple or complex ABTs, a generic recurrence risk estimation for unfavourable pregnancy outcomes is challenging, as rearrangements can be individually rare or even unique in each carrier/family [2,5]. Risk estimations mostly rely on empirical data [6–8]; however, they can be influenced by several factors, such as the sex of the parental carrier, the type and number of chromosomes involved in each ABT, the number and precise location of breakpoints, as well as the length of the chromosomal region involved in the rearrangement [2,9,10].

The phenotypic risk is thought to be very low in carriers of familial ABTs inherited from non-affected parents [11,12]; nevertheless, a number of ABT offspring with clinical phenotypes have been reported [11,13–15]. There are very limited studies investigating, in detail, the genetic causality of such discordant phenotypes [12,16]. We recently demonstrated the power of low-coverage, whole-genome mate-pair sequencing (WG-MPS) in precisely detecting ABT breakpoints in four families having both affected and non-affected individuals carrying the same apparently balanced rearrangements [12]. After thoroughly studying all possible mechanisms that could explain the differential phenotypes, it appeared that the investigated ABTs were identical and truly balanced in each family, and thus, unrelated to phenotype development [12]. In contrast, in de novo ABT cases and familial ABT cases with segregating phenotypes, the translocation itself frequently explains associated phenotypes through several mechanisms [17–19]. These include direct disruption of disease-associated genes [20–22], presence of cryptic imbalances and/or complexity at/near the translocation breakpoints [11,14,23,24], and long-range position effects altering the expression of disease-associated genes mapped in the vicinity of the ABT breakpoints [25–27].

Recent advances in whole exome sequencing (WES) offer a high-throughput, cost-effective method for detecting disease-associated single-nucleotide variants (SNVs) and insertion-deletions (indels) across all exonic regions in the genome, underlying Mendelian diseases [28–30]. In the present study, the same four ABT families were analysed using WES as a diagnostic tool to identify the underlying genetic aetiology of the patients' phenotypes. Based on our findings, novel candidate variants potentially explaining phenotypic differences, and occurring independently from the common familial translocations, were identified in three out of four families.

2. Materials and Methods

2.1. Study Participants

Four families, each having one affected and at least one non-affected carrier of identical ABTs [12], were followed-up in the present WES study. The patients' phenotypes, in Human Phenotype Ontology (HPO) terms [31], as well as previous karyotype analysis results, are presented in Table 1.

2.2. Whole Exome Sequencing

WES libraries for all samples included in the present study were prepared by using the Nextera Rapid Capture Exome kit (Illumina Inc., San Diego, CA, USA) according to the manufacturer's protocol (Nextera Rapid Capture Enrichment Reference Guide, Illumina, Document #15037436 v01). Paired-end sequencing of the pooled libraries was performed on a NextSeq 500 system (Illumina) by using the NextSeq 500/550 High Output Kit v2.5 (300 Cycles) and following the manufacturer's guidelines (NextSeq System Denature and Dilute Libraries Guide, Document #15048776, v02, and NextSeq System Guide, Illumina, Document #15046563, v02). Demultiplexing and adapter trimming was performed automatically using BaseSpace Sequencing Hub Apps (Illumina).

Table 1. List of families/samples included in the present study.

Family Number	Sample Name	Reason for Referral	Phenotypes (HPO)	Karyotype
1	1A	Affected individual with intellectual disability, psychomotor delay, epilepsy and multifocal epileptiform discharges	HP:0001249<br>HP:0001263<br>HP:0001250<br>HP:0010841	46,XY,t(1;7)(p36.1;q22)mat
1	1B	Mother of sample 1A	Non-affected	46,XX,t(1;7)(p36.1;q22)
1	1C	Father of sample 1A	Non-affected	46,XY
2	2A	Affected individual with severe intellectual disability and microcephaly	HP:0010864<br>HP:0000252	46,XX,t(7;8)(q32;q24.13)
2	2B	Sibling of sample 2A	Non-affected	46,XX,t(7;8)(q32;q24.13)
3	3A	Affected individual with mild intellectual disability, short stature, low set ears, flat mid face, long neck, high arched palate, and simian palmar crease	HP:0001256<br>HP:0004322<br>HP:0000369<br>HP:0011800<br>HP:0000472<br>HP:0000218<br>HP:0000954	46,XX,t(4;10)(q35;q11.2)mat
3	3B	Mother of sample 3A	Non-affected	46,XX,t(4;10)(q35;q11.2)
3	3C	Sibling of sample 3A	Non-affected	46,XX,t(4;10)(q35;q11.2)mat
4	4A	Affected individual with polysyndactyly and oral anomalies	HP:0001159<br>HP:0010442<br>HP:0000153	46,XY,t(1;20)(p35.3;q13.3)mat
4	4B	Mother of sample 4A	Non-affected	46,XX,t(1;20)(p35.3;q13.3)
4	4C	Father of sample 4A	Non-affected	46,XY

2.3. WES Data Processing and Variant Annotation

Initially, downstream WES data processing and analysis was manually performed using an in-house bioinformatics exome analysis pipeline, which was based on the Genome Analysis Toolkit (GATK) best practices (v3.3) (Broad Institute, Cambridge, MA) [32,33]. The analysis was performed on Cy-Tera High Performance Computing clusters of the Cyprus Institute. A detailed description of this in-house pipeline for the identification and annotation of SNVs, indels and CNVs is available within the Supplementary Materials (Document S1, Figure S1, Tables S1 and S2) [32,34–43].

A reanalysis was also performed using the VarSome Clinical platform (version 11.0.0) [44]. Reads, in FASTQ format, were loaded on the platform and were aligned to the human reference genome (hg19) using Sentieon (bwa-mem) aligner, while variant calling for SNVs and small indels was performed using Sentieon DNAscope caller. Variants were classified as pathogenic, likely pathogenic, variant of uncertain significance (VUS), likely benign, and benign, according to the American College of Medical Genetics and Genomics (ACMG) guidelines [45,46], the Association for Clinical Genomic Science (ACGS) recommendations [47], and assisted by VarSome's automated ACMG classifier (version 11.4.3). Variants were filtered according to the suspected inheritance pattern (algorithmic filtering), while only variants with a gnomAD frequency lower than 1% and 5% in de novo and recessive analyses, respectively, variants overlapping coding or splicing regions, and variants with coverage >8X were kept for further analysis. Furthermore, custom gene lists were created using patient phenotypes in HPO terms (Table 1), to retain disease-associated gene variants in each family. Additional and updated annotation available within VarSome Clinical, assisted the interpretation and selection of candidate variants for validation. Moreover, a CNV analysis was performed using the ExomeDepth tool [43] available within VarSome Clinical.

2.4. Candidate Variant Validation

All candidate variants were validated by conventional polymerase chain reaction (PCR) using Taq DNA Polymerase (QIAGEN, Hilden, Germany), followed by purification using the ExoSAP-IT™ PCR Product Cleanup Reagent (Applied Biosystems, Thermo Fisher Scientific, Waltham, MA, USA), bidirectional Sanger sequencing using the BigDye™ Terminator v1.1 Cycle Sequencing Kit (Applied Biosystems, Thermo Fisher Scientific), clean-up of the cycle sequencing reactions using Performa® DTR Gel Filtration Cartridges (EdgeBio, Gaithersburg, MD, USA), and capillary electrophoresis on a 3130xl Genetic Analyzer (Applied Biosystems, Thermo Fisher Scientific). Sanger sequencing data was analysed using the ABI Sequencing Analysis Software v5.4 (Applied Biosystems, Thermo Fisher Scientific). The PCR primers (Metabion, Planegg, Germany), flanking the candidate WES variants, were designed using the Primer 3 web tool, version 4.1.0 [48] (Table S3). The specific PCR and Sanger sequencing reaction volumes and conditions used are available upon request.

2.5. In Silico Analysis of WES Variants

In silico analysis to assess the impact of SNVs and indels, identified by WES, was performed using VarSome Clinical. The predicted pathogenicity of candidate variants was determined based on the combined evidence from multiple in silico predictors using the BayesDel_addAF meta-score. This ranged from −1.29334 to 0.75731; the higher the score, the more likely the variant was pathogenic. Apart from the automatic predictions by VarSome Clinical, the impact of candidate missense variants on protein structure and function was additionally assessed using Missense3D [49] and/or HOPE [50].

2.6. RNA Extraction

In order to further investigate a candidate splice-site variant identified by WES, RNA was extracted from Epstein-Barr virus transformed lymphoblastoid cell lines of the patient and a control sample using the RNeasy Midi Kit (QIAGEN), following the manufacturer's recommendations. On-column DNase digestion was performed using the RNase-Free DNase Set (QIAGEN) to ensure the removal of any residual DNA. The concentration and purity of the extracted RNA samples was measured using a NanoDrop™ 1000 Spectrophotometer (Thermo Fisher Scientific).

2.7. Reverse-Transcription PCR

DNase-treated RNA samples (1µg) were reverse transcribed to complementary DNA (cDNA) using the Protoscript® First Strand cDNA Synthesis Kit (New England Biolabs, Ipswich, MA, USA) and following the manufacturer's protocol. cDNA samples (1µg) from the patient and non-affected father (control sample), as well as the respective RNA samples not reverse transcribed (RT-ve) and a no-template (water) sample, were PCR amplified using HotStarTaq Plus DNA polymerase (QIAGEN) and custom reverse-transcription PCR (RT-PCR) primers (Metabion) (Table S4). The integrity of the cDNA samples was tested by amplification of a 215 bp fragment from the β-actin gene (*ACTB*) (Table S4). All RT-PCR products were loaded on a 2% agarose gel, which was pre-stained with GelRed® (1X final concentration) (Biotium, Fremont, CA, USA) and run at 120V for ~1 h. Selected RT-PCR products of interest were purified and further processed with bidirectional Sanger sequencing as described above. The specific RT-PCR reaction volumes and conditions used are available upon request.

2.8. Gene and Variant Nomenclature

Genes were described according to the HUGO Gene Nomenclature Committee (HGNC) guidelines [51]. All candidate variants were described according to the latest Human Genome Variation Society (HGVS) guidelines [52]. All genomic coordinates in the present article were based on the human reference genome hg19.

3. Results

Apparently balanced translocations were previously identified in all four families, having both affected and non-affected individuals [12]. After extensive workup, the genetic aetiology of the affected individuals remained unknown; therefore, WES was employed next to potentially unravel unidentified patient-specific candidate variants, in addition to the common ABTs in each family, and make genotype–phenotype correlations.

Depending on the availability of parental DNA samples, WES was performed in two patient-parent trios (families 1 and 4), one trio including the affected patient, non-affected mother, and non-affected sibling (family 3), and one duo including the affected patient and non-affected sibling (family 2). The in silico trio check (Document S1), applied for families 1 and 4, confirmed the child-parental relationships. Overall, the WES run was successful, with 90.06% of clusters passing filter, and a high base call accuracy, with 80.13% of bases having a Q score of 30. The mean target sequencing depth of the samples was ~75X, with more than 81% of target regions having at least 20X coverage, while the mean uniformity was 90.3%. Validated candidate variants, commonly identified by both the in-house analysis pipeline and the most recently annotated VarSome Clinical, are presented in Table 2 and described further below for each family.

Table 2. List of novel candidate variants identified by WES in each family. Candidate variant description is given according to the Human Genome Variation Society nomenclature recommendations. The criteria that apply in each case for pathogenicity classification are given. Allelic balance is defined here as the proportion of reads that support the variant, while coverage as the number of reads that align at the variant position with alignment quality ≥20.

Candidate Variant	Candidate Gene	Classification	MOI [1]	Sample Name [3]	Zygosity	Allelic Balance	Coverage
**Family 1:** NM_003165.6:c.1110+2T>G NP_003156.1:p.(Asp371Glyfs Ter16)	*STXBP1*	Pathogenic PVS1_very strong PS2_moderate PM2_suporting	AD [2]	1A	heterozygous	0.52	181
				1B	homozygous reference	0.0122	164
				1C	homozygous reference	0	140
**Family 2:** NM_006009.4:c.875C>T NP_006000.2:p.(Thr292Ile)	*TUBA1A*	Likely Pathogenic PM1_supporting PM2_supporting PP3_strong	AD [2]	2A	heterozygous	0.48	211
				2B	homozygous reference	0	225
**Family 3:** NM_006920.6:c.5060A>G NP_008851.3:p.(Glu1687Gly)	*SCN1A*	VUS PM1_moderate PM2_supporting PP3_moderate	AD [2]	3A	heterozygous	0.46	194
				3B	homozygous reference	0	231
				3C	homozygous reference	0	205

[1] MOI = mode of inheritance; [2] AD = autosomal dominant; [3] Please refer to Table 1.

3.1. Family 1

WES trio data analysis revealed a heterozygous splice donor variant NM_003165.6:c.1110+2T>G mapping to intron 13/19 of the syntaxin-binding protein 1 (*STXBP1*) gene (OMIM *602926) in the patient (sample 1A) (Figure 1A; Table 2). The specific splice donor variant was classified as pathogenic since it overlapped a gene for which loss-of-function is a known mechanism of disease. It was also absent from the gnomAD, VarSome Clinical and ClinVar databases, as well as our local database, thus making it a novel finding. In addition, multiple in silico tools used within VarSome Clinical predicted a damaging/pathogenic effect (BayesDel_addAF meta-score = 0.625).

Sanger sequencing confirmed that the identified splice donor variant occurred as a de novo event in the patient (Figure 1A). The impact of the NM_003165.6:c.1110+2T>G variant on *STXBP1* pre-mRNA splicing was also demonstrated using RT-PCR. Specifically, cDNA amplification using custom primers (*STXBP1*-11/12F and *STXBP1*-15R) flanking the splicing variant (Figure 1B) resulted in a single band in both the patient and the non-affected father (control) (Figure 1C). Bidirectional sequencing confirmed that both PCR products correspond to the wild-type *STXBP1* allele. However, cDNA amplification using *STXBP1*-mut13F, a primer specifically amplifying the mutated allele (Figure 1B), resulted in a PCR product only in the patient (Figure 1C), indicating the possible retention of intronic sequences into the mature *STXBP1* messenger RNA (mRNA). Indeed, subsequent sequencing of this product confirmed the addition of twenty-two intron 13 nucleotides in the *STXBP1* mRNA, followed by exon 14 sequences (Figure 1D). At the protein level, the splice donor variant was predicted to result in the insertion of fifteen new amino acids at position 371 of the STXBP1 protein followed by a premature termination codon (PTC) NP_003156.1:p.(Asp371GlyfsTer16) (Figure 1E).

3.2. Family 2

WES duo data analysis revealed a heterozygous patient-specific missense variant NM_006009.4:c.875C>T in exon 4/4 of the Tubulin α 1A (*TUBA1A*) gene (OMIM *602529) (Figure S2A; Table 2). This novel finding was classified as likely pathogenic, since it was not recorded in the gnomAD, VarSome Clinical, ClinVar or our local databases. Furthermore, it overlapped a mutational hot-spot, while multiple in silico tools used within VarSome Clinical predicted a damaging/pathogenic effect (BayesDel_addAF meta-score = 0.461).

Sanger sequencing confirmed that the affected sibling (sample 2A) was a heterozygous carrier of the mutant allele, while the non-affected sibling (sample 2B) was homozygous for the reference allele (Figure S2A); however, possible inheritance of this variant could not be established, as parental genomic samples were not available for testing. At the protein level, the identified missense variant was predicted to change the highly-conserved neutral amino acid threonine to the hydrophobic amino acid isoleucine at position 292 of the TUBA1A protein (NP_006000.2:p.(Thr292Ile)). Furthermore, a structural alteration was detected when the impact of the NP_006000.2:p.(Thr292Ile) variant was assessed with Missense3D and HOPE (Figure S2B). Specifically, the mutant isoleucine residue affects hydrogen bond formation between the wild-type threonine residue and other amino acids in the core of the TUBA1A protein, thus disturbing correct folding.

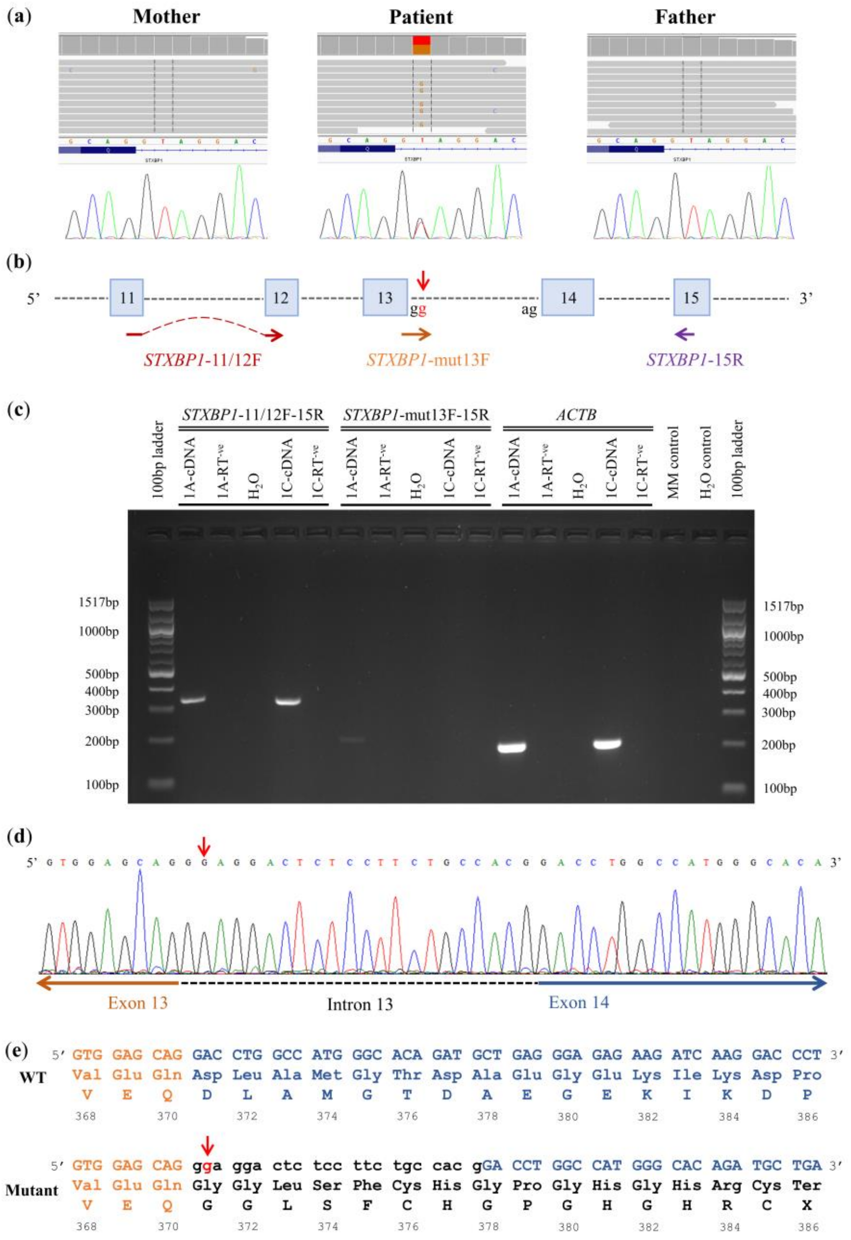

Figure 1. Family 1 candidate variant investigation. (**a**) Top panel: Integrative Genomics Viewer (IGV) screenshots of the genomic region overlapping the patient-specific *STXBP1* splice donor variant. Lower panel: Sanger sequencing confirming that the patient was heterozygous for the mutated allele (T/G), while both parents are homozygous for the reference allele (T/T). (**b**) Schematic representation of the *STXBP1* gene (exons 11–15) and reverse-transcription PCR (RT-PCR) primers used in this study: primer *STXBP1*-11/12F spans two consecutive exons to ensure that only cDNA will be amplified, primer *STXBP1*-15R maps downstream the splice-site variant on the proximal end of exon 15, while primer *STXBP1*-mut13F specifically amplifies the mutated allele (vertical red arrow). (**c**) Agarose gel electrophoresis of RT-PCR amplicons from the patient (sample 1A) and his non-affected father (sample 1C). The integrity of the cDNA samples is tested by amplification of an *ACTB* gene fragment. RNA samples not reverse transcribed (RT-ve) and no template (H_2O) samples are used as negative controls to detect any contaminating DNA. A master mix (MM) control is also included. (**d**) Impact of the NM_003165.6:c.1110+2T>G variant (red vertical arrow) at the RNA level; twenty-two intron 13 nucleotides are inserted between exons 13 and 14 in the mature mRNA of the patient. (**e**) Predicted impact of the NM_003165.6:c.1110+2T>G variant (red vertical arrow) at the protein level; fifteen new amino acids are included followed by a premature termination codon. Nucleotides from exon 13 and 14 sequences are presented in orange and blue, respectively, while intron 13 sequences are shown in black lower-case letters. Numbers correspond to amino acid positions according to NP_003156.1.

3.3. Family 3

WES multi-sample data analysis was initially focused on homozygous recessive variants in the patient (sample 3A), because of the reported consanguinity in the family (parents are first cousins); however, no significant findings were detected. Screening for heterozygous dominant variants in the patient revealed a heterozygous missense variant NM_006920.6:c.5060A>G in exon 29/29 of the Sodium Channel, Neuronal type I, α subunit (*SCN1A*) gene (OMIM *182389) (Figure S3A; Table 2). This was classified as VUS, as it was absent from publicly available databases and our local database, thus making it a novel finding. In addition, the identified *SCN1A* missense variant overlapped a mutational hotspot, while a number of in silico tools used within VarSome Clinical predicted a damaging/pathogenic effect (BayesDel_addAF meta-score = 0.298).

Sanger sequencing confirmed the heterozygous *SCN1A* variant in the affected proband (sample 3A), while the non-affected mother (sample 3B) and male sibling (sample 3C) were both homozygous for the reference allele (Figure S3B). However, as paternal genomic material was not available for testing, it was not confirmed yet whether this was a de novo variant. At the protein level, the identified *SCN1A* variant was predicted to change the hydrophilic Glutamic acid to the neutral amino acid Glycine at position 1687 of the SCN1A protein (NP_008851.3:p.(Glu1687Gly)) (Figure S3C). This could result in loss of hydrogen bonds and/or disturb correct protein folding.

3.4. Family 4

WES trio data analysis, with our in-house pipelines and the most recently annotated VarSome Clinical, revealed no pathogenic/likely-pathogenic SNVs or CNVs in the proband of family 4 that could explain the presented polysyndactyly and oral phenotypes.

4. Discussion

Based on our previous findings, translocations in the four ABT families included in the current study were considered coincidental and unrelated with the observed phenotypes [12]. As a next step, WES was implemented as a diagnostic tool to identify the underlying genetic aetiology of the patients' clinical phenotypes. Patient-specific candidate variants, which were missed by low-coverage WG-MPS and other previous analyses, were identified in three out of four families. These were discussed in further detail, as seen below.

In family 1, besides the non-pathogenic familial ABT t(1;7)(p36.1;q22) previously identified by WG-MPS [12], a novel heterozygous pathogenic *STXBP1* splice donor variant was revealed by WES in the patient. This gene is highly expressed in the brain and encodes the syntaxin-binding protein 1, which has an essential role in the regulation of vesicle trafficking, membrane fusion, and neurotransmitter release in neuronal synapses [53,54]. Several studies have previously identified pathogenic *STXBP1* SNVs [55–58], as well as partial or whole gene deletions [55,59] in patients with developmental and epileptic encephalopathy 4 (OMIM # 612164), overlapping with the symptoms—intellectual disability, global developmental delay, seizures and multifocal epileptiform discharges—seen in our patient. Variants affecting *STXBP1* splicing have also been reported before [56,57,60–64]. In the present study, RT-PCR results support that the novel de novo splice donor variant NM_003165.6:c.1110+2T>G affects *STXBP1* pre-mRNA splicing in the patient. Intron inclusion in the mature mRNA was predicted to result in the introduction of a PTC in exon 14, more than 50 to 55 nucleotides upstream from the last exon–exon junction, and was therefore targeted for nonsense mediated decay [65]. The truncated STXBP1 protein was predicted to be dysfunctional, as NP_003156.1:p.(Asp371GlyfsTer16) disrupts STXBP1 domain 3a which, together with domain 1, forms the central cavity of STXBP1 for syntaxin binding, an important step for neurotransmitter release in the synaptic cleft [66]. Overall, the identified de novo *STXBP1* splice donor variant segregated with the patient's phenotype based on the biological

function of the gene, previous reports of *STXBP1* splicing variants in similar patients, and supporting RT-PCR results from the present study, thus expanding the mutational spectrum of the *STXBP1* gene.

In family 2, both siblings were carriers of a familial ABT, t(7;8)(q32;q24.13), which apparently was coincidental and did not contribute to phenotype development [12]. Subsequent WES analysis revealed a novel heterozygous likely pathogenic *TUBA1A* missense variant in the affected sibling, presenting with severe intellectual disability and microcephaly, which was absent from the non-affected sibling. The *TUBA1A* gene, highly expressed in brain, encodes for tubulin α 1A, which together with β tubulin, constitute the main components of microtubules as a heterodimer complex [67]. Microtubules play an important role in many cellular processes, including neuronal maturation and migration; when these are impaired, a wide spectrum of brain malformations, known as tubulinopathies, develop [55]. Heterozygous missense variants in the *TUBA1A* gene have been reported in patients with lissencephaly 3 (OMIM # 611603) [68–70]; a cortical malformation caused by neuronal migration defects and characterized by smooth brain surface. Associated clinical phenotypes include microcephaly and severe-to-profound intellectual disability, resembling those seen in our patient, as well as hypotonia, which was not present in our patient. The vast majority of pathogenic or likely pathogenic *TUBA1A* gene variants reported so far have been de novo heterozygous missense variants [71], with very few cases having *TUBA1A* nonsense variants, frameshifts, or deletions, indicating that such variants could have higher functional impacts, leading more severe phenotypes or even lethality. Dominant-negative disruption of microtubule formation rather than haploinsufficiency was thus suggested as the mechanism underlying *TUBA1A* variants [70,72]. Even though the de novo occurrence of the identified *TUBA1A* variant NM_006009.4:c.875C>T could not be determined and lissencephaly could not be confirmed in our patient due to the unavailability of an MRI scan, there was still enough evidence suggesting that it could be causative for the patient's phenotype. Future *TUBA1A* expression and/or functional analyses will further support such positive associations.

In family 3, WES analysis in the affected proband, non-affected mother, and non-affected male sibling, all sharing a common t(4;10)(q35;q11.2) rearrangement [12], identified a novel heterozygous patient-specific *SCN1A* missense variant of uncertain significance. The *SCN1A* gene encodes the neuronal sodium voltage-gated channel α subunit 1 (Nav1.1) that is composed of four homologous six-transmembrane segments [73]. SCN1A, together with two β subunits, forms the complete voltage-gated sodium channel, which is important for action potential generation and propagation in neurons [73]. Heterozygous *SCN1A* SNVs and CNVs have been previously reported in patients with intellectual disability, developmental delay, and a spectrum of epileptic encephalopathies of varied severity [74–76], ranging from the milder form of generalized epilepsy with febrile seizures plus type 2 (OMIM # 604403)-to-severe myoclonic epilepsy of infancy (or Dravet Syndrome) (OMIM # 607208) [77,78]. According to the referring doctor, patient 3A was 43 years old at the time of study and did not have epileptic episodes. Clinical severity variability in individuals with *SCN1A* disruptions depends, not only on the type, but also the location of the specific variants [79,80]. In addition, it has been suggested that mosaicism [81], as well as other environmental and/or genetic factors, such as variants in modifier genes, could influence clinical presentation and thus explain variable phenotypes in individuals with de novo or inherited *SCN1A* variants [75,82]. It was, therefore, extremely important to investigate the functional impact and mechanism of each identified *SCN1A* variant to explain the severity of clinical manifestations [83]. Due to consanguinity in the family, identification of a recessive variant would have been expected in the patient; however, no such candidate variant was revealed that could explain the presented phenotype. At this stage, the patient-specific *SCN1A* variant seemed to be the most promising candidate; however, a strong causative role could not yet be established for this VUS. Revisiting the medical history of the patient and complementary

functional studies will be required in order to strengthen such associations. Alternatively, careful reevaluation of the family with other methods, as suggested further below, could be done to possibly identify non-coding SNVs, variants within repetitive regions, or cryptic rearrangements that could have been missed by WES.

The patient in family 4 presented with polysyndactyly and oral anomalies which, upon clinical evaluation, were indicative for an orofaciodigital syndrome (OFDS). OFDS are a group of highly heterogeneous ciliopathy disorders characterized predominantly by abnormalities in the mouth, face, and digits [84]. There are at least fourteen OFDS subtypes, with overlapping clinical manifestations, as well as two rare unclassified ones [85]. The majority of known causal genes for OFDS encode proteins mapping in cilia compartments and regulating ciliogenesis [85]; however, novel genes and variants are continuously discovered with the use of NGS-based methods [86,87]. Furthermore, OFDS genes and clinical features are also implicated in other ciliopathies, such as Joubert syndrome and Bardet–Biedl syndrome [85,86]. In family 4, WES data was filtered to retain variant disrupting genes involved in OFDS, Joubert syndrome and polysyndactyly to cover potential differential diagnoses. However, no candidate variants could be identified, from the in-house bioinformatics pipeline or VarSome Clinical analysis, that could explain the patient's polysyndactyly and oral anomalies. Therefore, detailed clinical examination of the patient, together with careful reevaluation of the family's WES data, and further investigation using alternative NGS-based methods, as described below, will be necessary to potentially resolve this case.

Most of the disease-causing variants reside in the exome, thus making WES a popular sequencing approach in clinical diagnostics and human genetics research [88,89]. While WES has significant diagnostic potential, it also has certain limitations that could hinder such insightful discoveries [90]. First of all, WES has a limited ability to identify clinically-relevant variants outside the targeted capture region; thus, high-coverage whole genome sequencing, assessing both coding and non-coding regions, should be considered to potentially increase the diagnostic yield [91,92]. Furthermore, WES does not adequately cover the entire exome; regions with high GC content are particularly difficult to capture and sequences can result in few or no mappable reads. Meanwhile, short-reads in highly-repetitive regions fail to align unambiguously, leading to low mapping quality issues [93]. Consequently, clinically-relevant gene variants (e.g., repeat expansions) and SVs overlapping such regions could be easily missed using short-read sequencing technologies. Therefore, long-read sequencing approaches offering a more uniform genome-wide coverage, such as nanopore sequencing by Oxford Nanopore Technologies and single molecule real-time sequencing by PacBio, should be considered as alternative approaches [94,95]. Finally, unresolved ABT families with discordant phenotypes could also be studied with Bionano Optical Genome Mapping, a next-generation cytogenomics tool that could potentially detect patient-specific cryptic CNVs and complexities missed by other methods [96].

5. Conclusions

The current study presented a follow-up investigation of four familial ABTs, where WES was implemented as a diagnostic tool to identify the underlying genetic aetiology of these patients' clinical phenotypes. Novel candidate variants were identified in three out of four families. Regarding the pathogenic *STXBP1* splicing variant and likely pathogenic *TUBA1A* missense variant identified in Families 1 and 2, respectively, there was strong evidence to support the underlying genetic cause of the patients' phenotype. The *SCN1A* missense variant identified in family 3 was of uncertain significance and will require additional clinical and molecular evidence to sufficiently support causality. Based on our findings, WES can be recommended as a complementary test in familial ABT cases where cryptic complexity has been excluded. We anticipate that careful reevaluation of unsolved families, along with investigation of additional ABT families with discordant phenotypes, will further support our findings and help to decipher the underlying genetic

mechanisms—as well as provide more precise phenotypic risk estimations and better genetic counselling.

Supplementary Materials: The following supporting information can be downloaded at: https://www.mdpi.com/article/10.3390/genes14010082/s1, Document S1: In-house Bioinformatic Exome Analysis Pipeline and Supplementary Figures; Figure S1: In-house bioinformatics pipeline for variant calling of an exome sequencing paired-end experiment using the Illumina NextSeq500 platform.; Figure S2: Family 2 candidate variant investigation. (a) Top panel: IGV screenshot depicting the patient-specific *TUBA1A* missense variant identified by WES. Lower panel: Sanger sequencing confirmed that the affected sibling (sample 2A) was heterozygous for the mutated allele (red arrow), while the non-affected sibling (sample 2B) was homozygous for the reference allele; (b) Top panel: Schematic structures of the wild-type threonine (left) and the mutant isoleucine (right) amino acids. The backbone, which is the same for each amino acid, is coloured red. The side chain, unique for each amino acid, is coloured black. Lower panel: Structural analysis of the NP_006000.2:p.(Thr292Ile) variant (Protein Data Bank code: 5JCO); the mutant isoleucine (right) affects hydrogen bonds formed by the wild-type threonine (blue), thus disturbing correct protein folding.; Figure S3: Family 3 candidate variant investigation. (a) IGV screenshot depicting the patient-specific *SCN1A* candidate variant identified by WES; (b) Sanger sequencing confirmed that the proband of family 3 (sample 3A) is heterozygous for the mutated allele (red arrow), while the non-affected mother (sample 3B) and male sibling (sample 3C) are both homozygous for the reference allele; (c) Schematic structures of the wild-type glutamic acid (left) and the mutant glycine (right) amino acids. The backbone, which is the same for each amino acid, is coloured red. The side chain, unique for each amino acid, is coloured black.; Table S1: List of genes included in the Greenwood Genetic Center X-linked intellectual disability gene panel used for WES data filtering in ABT families 1, 2, and 3.; Table S2: List of genes associated with polysyndactyly/synpolydactyly, orofaciodigital syndrome, and Joubert syndrome used for WES data filtering in ABT family 4.; Table S3: List of PCR primers used to validate candidate WES variants in each ABT family.; Table S4: List of Reverse-Transcription PCR primers used to investigate the underlying mechanism of the identified *STXBP1* splice donor variant in family 1.

Author Contributions: Conceptualization, C.S.; methodology, C.A., A.T. and C.S.; software, A.T.; validation, A.A. and I.P.; formal analysis, A.T. and C.A.; investigation, C.A., A.T., A.A. and I.P.; resources, C.S., P.E., Z.K.-A., F.B., V.C.-A. and G.A.T.; data curation, A.T. and C.A.; writing—original draft preparation, C.A. and A.T.; writing—review and editing, C.A., A.T. and C.S.; visualization, C.S., C.A. and A.T.; supervision, C.S. and P.E.; project administration, C.S.; funding acquisition, C.S. and C.A. All authors have read and agreed to the published version of the manuscript.

Funding: This research was funded by Telethon Cyprus and Norway Grants through the Directorate General for European Programmes, Coordination and Development of the Republic of Cyprus. WES data analysis was supported by the Cy-Tera Project (NEA ΥΠΟΔΟΜΗ/ΣΤΡΑΤΗ/0308/31), which is co-funded by the European Regional Development Fund and the Republic of Cyprus through the Cyprus Research and Innovation Foundation.

Institutional Review Board Statement: The study was conducted in accordance with the Declaration of Helsinki, and approved by the Cyprus National Bioethics Committee as part of the Translation Facility Application (EEBK/EII/2-13/09).

Informed Consent Statement: Informed consent was obtained from all subjects involved in the study.

Data Availability Statement: All sequence variants presented in this study are available from the ClinVar database (https://www.ncbi.nlm.nih.gov/clinvar) with accession numbers SCV002757998-SCV002758000. The remaining data generated or analysed during this study are included within the published article and its supplementary files.

Acknowledgments: The authors would like to sincerely thank the patients and their families for participating in the study. We also thank Charithea Ioannidou for her technical assistance, and Ludmila Kousoulidou for her help with reviewing this manuscript.

Conflicts of Interest: The authors declare no conflict of interest.

References

1. Scriven, P.N.; Handyside, A.H.; Ogilvie, C.M. Chromosome translocations: segregation modes and strategies for preimplantation genetic diagnosis. *Prenat. Diagn.* **1998**, *18*, 1437–1449.
2. Madan, K. Balanced complex chromosome rearrangements: Reproductive aspects. A review. *Am. J. Med. Genet. A.* **2012**, *158A*, 947-963. https://doi.org/10.1002/ajmg.a.35220.
3. Warburton, D. De novo balanced chromosome rearrangements and extra marker chromosomes identified at prenatal diagnosis: clinical significance and distribution of breakpoints. *Am. J. Hum. Genet.* **1991**, *49*, 995–1013.
4. Halgren, C.; Nielsen, N.M.; Nazaryan-Petersen, L.; Silahtaroglu, A.; Collins, R.L.; Lowther, C.; Kjaergaard, S.; Frisch, M.; Kirchhoff, M.; Brøndum-Nielsen, K.; et al. Risks and Recommendations in Prenatally Detected De Novo Balanced Chromosomal Rearrangements from Assessment of Long-Term Outcomes. *Am. J. Hum. Genet.* **2018**, *102*, 1090–1103. https://doi.org/10.1016/j.ajhg.2018.04.005.
5. Young, I.D. *Introduction to Risk Calculation in Genetic Counseling*; Oxford University Press: Oxford, UK, 2007.
6. Stengel-Rutkowski, S.; Stene, J.; Gallano, P. *Risk Estimates in Balanced Parental Reciprocal Translocations: Analysis of 1120 Pedigrees*; Expansion Scientifique Francaise: Paris, France, 1988.
7. Gorski, J.L.; Kistenmacher, M.L.; Punnett, H.H.; Zackai, E.H.; Emanuel, B.S.; Optiz, J.M.; Reynolds, J.F. Reproductive risks for carriers of complex chromosome rearrangements: Analysis of 25 families. *Am. J. Med. Genet.* **1988**, *29*, 247–261. https://doi.org/10.1002/ajmg.1320290202.
8. Neri, G.; Serra, A.; Campana, M.; Tedeschi, B. Reproductive risks for translocation carriers: cytogenetic study and analysis of pregnancy outcome in 58 families. *Am. J. Med. Genet.* **1983**, *16*, 535–561. https://doi.org/10.1002/AJMG.1320160412.
9. Stene, J.; Stengel-Rutowski, S. Genetic risks for familial reciprocal translocations with special emphasis on those leading to 9p, 10p and 12p trisomies. *Ann. Hum. Genet.* **1982**, *46*, 41–74. https://doi.org/10.1111/J.1469-1809.1982.TB00694.X.
10. Midro, A.T.; Stengel-Rutkowski, S.; Stene, J. Experiences with risk estimates for carriers of chromosomal reciprocal translocations. *Clin. Genet.* **1992**, *41*, 113–122. https://doi.org/10.1111/J.1399-0004.1992.TB03646.X.
11. Sismani, C.; Kitsiou-Tzeli, S.; Ioannides, M.; Christodoulou, C.; Anastasiadou, V.; Stylianidou, G.; Papadopoulou, E.; Kanavakis, E.; Kosmaidou-Aravidou, Z.; Patsalis, P.C. Cryptic genomic imbalances in patients with de novo or familial apparently balanced translocations and abnormal phenotype. *Mol. Cytogenet.* **2008**, *1*, 15–15. https://doi.org/10.1186/1755-8166-1-15.
12. Aristidou, C.; Koufaris, C.; Theodosiou, A.; Bak, M.; Mehrjouy, M.M.; Behjati, F.; Tanteles, G.; Christophidou-Anastasiadou, V.; Tommerup, N.; Sismani, C. Accurate breakpoint mapping in apparently balanced translocation families with discordant phenotypes using whole genome mate-pair sequencing. *PLoS ONE* **2017**, *12*, e0169935-e0169935. https://doi.org/10.1371/journal.pone.0169935.
13. Wenger, S.L.; Steele, M.W.; Boone, L.Y.; Lenkey, S.G.; Cummins, J.H.; Chen, X.Q. "Balanced" karyotypes in six abnormal offspring of balanced reciprocal translocation normal carrier parents. *Am. J. Med. Genet.* **1995**, *55*, 47–52. https://doi.org/10.1002/ajmg.1320550114.
14. Schluth-Bolard, C.; Delobel, B.; Sanlaville, D.; Boute, O.; Cuisset, J.-M.M.; Sukno, S.; Labalme, A.; Duban-Bedu, B.n.d.B.; Plessis, G.; Jaillard, S.; et al. Cryptic genomic imbalances in de novo and inherited apparently balanced chromosomal rearrangements: Array CGH study of 47 unrelated cases. *Eur. J. Med. Genet.* **2009**, *52*, 291–296. https://doi.org/10.1016/j.ejmg.2009.05.011.
15. Rao, L.; Kanakavalli, M.; Padmalatha, V.; Nallari, P.; Singh, L. Paternally derived translocation t(8;18)(q22.1;q22)pat associated in a patient with developmental delay: Case report and review. *J. Pediatr. Neurosci.* **2010**, *5*, 64–67. https://doi.org/10.4103/1817-1745.66686.
16. Utami, K.H.; Hillmer, A.M.; Aksoy, I.; Chew, E.G.; Teo, A.S.; Zhang, Z.; Lee, C.W.; Chen, P.J.; Seng, C.C.; Ariyaratne, P.N.; et al. Detection of chromosomal breakpoints in patients with developmental delay and speech disorders. *PLoS ONE* **2014**, *9*, e90852-e90852. https://doi.org/10.1371/journal.pone.0090852.
17. Aristidou, C.; Theodosiou, A.; Bak, M.; Mehrjouy, M.M.; Constantinou, E.; Alexandrou, A.; Papaevripidou, I.; Christophidou-Anastasiadou, V.; Skordis, N.; Kitsiou-Tzeli, S.; et al. Position effect, cryptic complexity, and direct gene disruption as disease mechanisms in de novo apparently balanced translocation cases. *PLoS ONE* **2018**, *13*, e0205298-e0205298. https://doi.org/10.1371/journal.pone.0205298.
18. Redin, C.; Brand, H.; Collins, R.L.; Kammin, T.; Mitchell, E.; Hodge, J.C.; Hanscom, C.; Pillalamarri, V.; Seabra, C.M.; Abbott, M.-A.; et al. The genomic landscape of balanced cytogenetic abnormalities associated with human congenital anomalies. *Nat. Genet.* **2017**, *49*, 36–45. https://doi.org/10.1038/ng.3720.
19. Schluth-Bolard, C.; Diguet, F.; Chatron, N.; Rollat-Farnier, P.A.; Bardel, C.; Afenjar, A.; Amblard, F.; Amiel, J.; Blesson, S.; Callier, P.; et al. Whole genome paired-end sequencing elucidates functional and phenotypic consequences of balanced chromosomal rearrangement in patients with developmental disorders. *J. Med. Genet.* **2019**, *56*, 526–535. https://doi.org/10.1136/JMEDGENET-2018-105778.
20. Bugge, M.; Bruun-Petersen, G.; Brøndum-Nielsen, K.; Friedrich, U.; Hansen, J.; Jensen, G.; Jensen, P.K.; Kristoffersson, U.; Lundsteen, C.; Niebuhr, E.; et al. Disease associated balanced chromosome rearrangements: a resource for large scale genotype-phenotype delineation in man. *J. Med. Genet.* **2000**, *37*, 858–865. https://doi.org/10.1136/jmg.37.11.858.
21. Schluth-Bolard, C.; Labalme, A.; Cordier, M.-P.P.; Till, M.; Nadeau, G.; Tevissen, H.; Lesca, G.; Boutry-Kryza, N.; Rossignol, S.; Rocas, D.; et al. Breakpoint mapping by next generation sequencing reveals causative gene disruption in patients carrying apparently balanced chromosome rearrangements with intellectual deficiency and/or congenital malformations. *J. Med. Genet.* **2013**, *50*, 144–150. https://doi.org/10.1136/jmedgenet-2012-101351.

22. Nilsson, D.; Pettersson, M.; Gustavsson, P.; Förster, A.; Hofmeister, W.; Wincent, J.; Zachariadis, V.; Anderlid, B.-M.; Nordgren, A.; Mäkitie, O.; et al. Whole-Genome Sequencing of Cytogenetically Balanced Chromosome Translocations Identifies Potentially Pathological Gene Disruptions and Highlights the Importance of Microhomology in the Mechanism of Formation. *Hum. Mutat.* **2017**, *38*, 180–192. https://doi.org/10.1002/humu.23146.
23. Patsalis, P.C.; Evangelidou, P.; Charalambous, S.; Sismani, C. Fluorescence in situ hybridization characterization of apparently balanced translocation reveals cryptic complex chromosomal rearrangements with unexpected level of complexity. *Eur. J. Hum. Genet.* **2004**, *12*, 647–653. https://doi.org/10.1038/sj.ejhg.5201211.
24. Gribble, S.M.; Prigmore, E.; Burford, D.C.; Porter, K.M.; Ng, B.L.; Douglas, E.J.; Fiegler, H.; Carr, P.; Kalaitzopoulos, D.; Clegg, S.; et al. The complex nature of constitutional de novo apparently balanced translocations in patients presenting with abnormal phenotypes. *J. Med. Genet.* **2005**, *42*, 8–16. https://doi.org/10.1136/jmg.2004.024141.
25. Kleinjan, D.-J.; van Heyningen, V. Position Effect in Human Genetic Disease. *Hum. Mol. Genet.* **1998**, *7*, 1611–1618. https://doi.org/10.1093/hmg/7.10.1611.
26. Velagaleti, G.V.N.; Bien-Willner, G.A.; Northup, J.K.; Lockhart, L.H.; Hawkins, J.C.; Jalal, S.M.; Withers, M.; Lupski, J.R.; Stankiewicz, P. Position Effects Due to Chromosome Breakpoints that Map ~900 Kb Upstream and ~1.3 Mb Downstream of SOX9 in Two Patients with Campomelic Dysplasia. *Am. J. Hum. Genet.* **2005**, *76*, 652–662. https://doi.org/10.1086/429252.
27. Finelli, P.; Sirchia, S.M.; Masciadri, M.; Crippa, M.; Recalcati, M.P.; Rusconi, D.; Giardino, D.; Monti, L.; Cogliati, F.; Faravelli, F.; et al. Juxtaposition of heterochromatic and euchromatic regions by chromosomal translocation mediates a heterochromatic long-range position effect associated with a severe neurological phenotype. *Mol. Cytogenet.* **2012**, *5*, 16–16. https://doi.org/10.1186/1755-8166-5-16.
28. Choi, M.; Scholl, U.I.; Ji, W.; Liu, T.; Tikhonova, I.R.; Zumbo, P.; Nayir, A.; Bakkaloğlu, A.; Özen, S.; Sanjad, S.; et al. Genetic diagnosis by whole exome capture and massively parallel DNA sequencing. *Proc. Natl. Acad. Sci. USA* **2009**, *106*, 19096–19096. https://doi.org/10.1073/PNAS.0910672106.
29. Ng, S.B.; Buckingham, K.J.; Lee, C.; Bigham, A.W.; Tabor, H.K.; Dent, K.M.; Huff, C.D.; Shannon, P.T.; Jabs, E.W.; Nickerson, D.A.; et al. Exome sequencing identifies the cause of a Mendelian disorder. *Nat. Genet.* **2010**, *42*, 30–30. https://doi.org/10.1038/NG.499.
30. Goh, G.; Choi, M. Application of Whole Exome Sequencing to Identify Disease-Causing Variants in Inherited Human Diseases. *Genomics Inform.* **2012**, *10*, 214–214. https://doi.org/10.5808/GI.2012.10.4.214.
31. Robinson, P.N.; Köhler, S.; Bauer, S.; Seelow, D.; Horn, D.; Mundlos, S. The Human Phenotype Ontology: A Tool for Annotating and Analyzing Human Hereditary Disease. *Am. J. Hum. Genet.* **2008**, *83*, 610–610. https://doi.org/10.1016/J.AJHG.2008.09.017.
32. McKenna, A.; Hanna, M.; Banks, E.; Sivachenko, A.; Cibulskis, K.; Kernytsky, A.; Garimella, K.; Altshuler, D.; Gabriel, S.; Daly, M.; et al. The Genome Analysis Toolkit : A MapReduce framework for analyzing next-generation DNA sequencing data. *Genome Res.* **2010**, *20*, 1297–1303. https://doi.org/10.1101/gr.107524.110.20.
33. Van der Auwera, G.A.; Carneiro, M.O.; Hartl, C.; Poplin, R.; del Angel, G.; Levy-Moonshine, A.; Jordan, T.; Shakir, K.; Roazen, D.; Thibault, J.; et al. From FastQ data to high confidence variant calls: the Genome Analysis Toolkit best practices pipeline. *Curr. Protoc. Bioinform.* **2013**, *11*, 11.10.11–11.10.11. https://doi.org/10.1002/0471250953.BI1110S43.
34. McLaren, W.; Gil, L.; Hunt, S.E.; Riat, H.S.; Ritchie, G.R.S.; Thormann, A.; Flicek, P.; Cunningham, F. The Ensembl Variant Effect Predictor. *Genome Biol.* **2016**, *17*, 122. https://doi.org/10.1186/s13059-016-0974-4.
35. Paila, U.; Chapman, B.A.; Kirchner, R.; Quinlan, A.R. GEMINI: Integrative Exploration of Genetic Variation and Genome Annotations. *PLoS Comput. Biol.* **2013**, *9*, e1003153. https://doi.org/10.1371/journal.pcbi.1003153.
36. Martin, M. Cutadapt removes adapter sequences from high-throughput sequencing reads. *EMBNet J. 2011* **2011**, *17*, 3. https://doi.org/10.14806/ej.17.1.200.
37. Li, H.; Durbin, R. Fast and accurate short read alignment with Burrows-Wheeler transform. *Bioinformatics* **2009**, *25*, 1754–1760. https://doi.org/10.1093/bioinformatics/btp324.
38. Li, H.; Handsaker, B.; Wysoker, A.; Fennell, T.; Ruan, J.; Homer, N.; Marth, G.; Abecasis, G.; Durbin, R.; Genome Project Data Processing Subgroup, G.P.D.P. The Sequence Alignment/Map format and SAMtools. *Bioinformatics* **2009**, *25*, 2078–2079. https://doi.org/10.1093/bioinformatics/btp352.
39. Pedersen, B.S.; Quinlan, A.R. Who's Who? Detecting and Resolving Sample Anomalies in Human DNA Sequencing Studies with Peddy. *Am. J. Hum. Genet.* **2017**, *100*, 406–413. https://doi.org/10.1016/J.AJHG.2017.01.017.
40. Schwarz, J.M.; Cooper, D.N.; Schuelke, M.; Seelow, D. MutationTaster2: mutation prediction for the deep-sequencing age. *Nat. Methods* **2014**, *11*, 361–362. https://doi.org/10.1038/nmeth.2890.
41. Desmet, F.O.; Hamroun, D.; Lalande, M.; Collod-Bëroud, G.; Claustres, M.; Béroud, C. Human Splicing Finder: An online bioinformatics tool to predict splicing signals. *Nucleic Acids Res.* **2009**, *37*, 1–14. https://doi.org/10.1093/nar/gkp215.
42. Vissers, L.E.L.M.; Gilissen, C.; Veltman, J.A. Genetic studies in intellectual disability and related disorders. *Nat. Rev. Genet.* **2015**, *17*, 9–18. https://doi.org/10.1038/nrg3999.
43. Plagnol, V.; Curtis, J.; Epstein, M.; Mok, K.Y.; Stebbings, E.; Grigoriadou, S.; Wood, N.W.; Hambleton, S.; Burns, S.O.; Thrasher, A.J.; et al. A robust model for read count data in exome sequencing experiments and implications for copy number variant calling. *Bioinformatics* **2012**, *28*, 2747–2754. https://doi.org/10.1093/BIOINFORMATICS/BTS526.
44. Kopanos, C.; Tsiolkas, V.; Kouris, A.; Chapple, C.E.; Albarca Aguilera, M.; Meyer, R.; Massouras, A. VarSome: the human genomic variant search engine. *Bioinformatics* **2019**, *35*, 1978–1980. https://doi.org/10.1093/BIOINFORMATICS/BTY897.

45. Richards, C.S.; Bale, S.; Bellissimo, D.B.; Das, S.; Grody, W.W.; Hegde, M.R.; Lyon, E.; Ward, B.E. ACMG recommendations for standards for interpretation and reporting of sequence variations: Revisions 2007. *Genet. Med.* **2008**, *10*, 294–300. https://doi.org/10.1097/GIM.0B013E31816B5CAE.
46. Richards, S.; Aziz, N.; Bale, S.; Bick, D.; Das, S.; Gastier-Foster, J.; Grody, W.W.; Hegde, M.; Lyon, E.; Spector, E.; et al. Standards and Guidelines for the Interpretation of Sequence Variants: A Joint Consensus Recommendation of the American College of Medical Genetics and Genomics and the Association for Molecular Pathology. *Genet. Med.* **2015**, *17*, 405–405. https://doi.org/10.1038/GIM.2015.30.
47. Ellard, S.; Baple, E.L.; Callaway, A.; Berry, I.; Forrester, N.; Turnbull, C.; Owens, M.; Eccles, D.M.; Abbs, S.; Scott, R.; et al. ACGS Best Practice Guidelines for Variant Classification in Rare Disease 2020. Available online: https://www.acgs.uk.com/media/11631/uk-practice-guidelines-for-variant-classification-v4-01-2020.pdf (accessed on 25 October 2022).
48. Rozen, S.; Skaletsky, H. Primer3 on the WWW for general users and for biologist programmers. *Methods Mol. Biol.* **2000**, *132*, 365–386. https://doi.org/10.1385/1-59259-192-2:365.
49. Ittisoponpisan, S.; Islam, S.A.; Khanna, T.; Alhuzimi, E.; David, A.; Sternberg, M.J.E. Can Predicted Protein 3D Structures Provide Reliable Insights into whether Missense Variants Are Disease Associated? *J. Mol. Biol.* **2019**, *431*, 2197–2212. https://doi.org/10.1016/j.jmb.2019.04.009.
50. Venselaar, H.; Te Beek, T.A.; Kuipers, R.K.; Hekkelman, M.L.; Vriend, G. Protein structure analysis of mutations causing inheritable diseases. An e-Science approach with life scientist friendly interfaces. *BMC Bioinform.* **2010**, *11*, 548. https://doi.org/10.1186/1471-2105-11-548.
51. Tweedie, S.; Braschi, B.; Gray, K.; Jones, T.E.M.; Seal, R.L.; Yates, B.; Bruford, E.A. Genenames.org: the HGNC and VGNC resources in 2021. *Nucleic Acids Res.* **2021**, *49*, D939-D939. https://doi.org/10.1093/NAR/GKAA980.
52. den Dunnen, J.T.; Dalgleish, R.; Maglott, D.R.; Hart, R.K.; Greenblatt, M.S.; McGowan-Jordan, J.; Roux, A.F.; Smith, T.; Antonarakis, S.E.; Taschner, P.E.M. HGVS Recommendations for the Description of Sequence Variants: 2016 Update. *Hum. Mutat.* **2016**, *37*, 564–569. https://doi.org/10.1002/HUMU.22981.
53. Burkhardt, P.; Hattendorf, D.A.; Weis, W.I.; Fasshauer, D. Munc18a controls SNARE assembly through its interaction with the syntaxin N-peptide. *EMBO J.* **2008**, *27*, 923–933, doi:emboj200837 [pii]\r10.1038/emboj.2008.37.
54. Han, G.A.; Malintan, N.T.; Collins, B.M.; Meunier, F.A.; Sugita, S. Munc18-1 as a key regulator of neurosecretion. *J. Neurochem.* **2010**, *115*, 1–10. https://doi.org/10.1111/j.1471-4159.2010.06900.x.
55. Saitsu, H.; Kato, M.; Mizuguchi, T.; Hamada, K.; Osaka, H.; Tohyama, J.; Uruno, K.; Kumada, S.; Nishiyama, K.; Nishimura, A.; et al. De novo mutations in the gene encoding STXBP1 (MUNC18-1) cause early infantile epileptic encephalopathy. *Nat. Genet.* **2008**, *40*, 782–788. https://doi.org/10.1038/ng.150.
56. Saitsu, H.; Kato, M.; Okada, I.; Orii, K.E.; Higuchi, T.; Hoshino, H.; Kubota, M.; Arai, H.; Tagawa, T.; Kimura, S.; et al. STXBP1 mutations in early infantile epileptic encephalopathy with suppression-burst pattern. *Epilepsia* **2010**, *51*, 2397–2405. https://doi.org/10.1111/j.1528-1167.2010.02728.x.
57. Deprez, L.; Weckhuysen, S.; Holmgren, P.; Suls, A.; Van Dyck, T.; Goossens, D.; Del-Favero, J.; Jansen, A.; Verhaert, K.; Lagae, L.; et al. Clinical spectrum of early-onset epileptic encephalopathies associated with STXBP1 mutations. *Neurology* **2010**, *75*, 1159–1165. https://doi.org/10.1212/WNL.0b013e3181f4d7bf.
58. Barcia, G.; Chemaly, N.; Gobin, S.; Milh, M.; Van Bogaert, P.; Barnerias, C.; Kaminska, A.; Dulac, O.; Desguerre, I.; Cormier, V.; et al. Early epileptic encephalopathies associated with STXBP1 mutations: Could we better delineate the phenotype? *Eur. J. Med. Genet.* **2014**, *57*, 15–20. https://doi.org/10.1016/j.ejmg.2013.10.006.
59. Saitsu, H.; Kato, M.; Shimono, M.; Senju, A.; Tanabe, S.; Kimura, T.; Nishiyama, K.; Yoneda, Y.; Kondo, Y.; Tsurusaki, Y.; et al. Association of genomic deletions in the STXBP1 gene with Ohtahara syndrome. *Clin. Genet.* **2012**, *81*, 399–402. https://doi.org/10.1111/j.1399-0004.2011.01733.x.
60. Hamdan, F.F.; Piton, A.; Gauthier, J.; Lortie, A.; Dubeau, F.; Dobrzeniecka, S.; Spiegelman, D.; Noreau, A.; Pellerin, S.; Côté, M.; et al. De novo STXBP1 mutations in mental retardation and nonsyndromic epilepsy. *Ann. Neurol.* **2009**, *65*, 748–753. https://doi.org/10.1002/ana.21625.
61. Saitsu, H.; Hoshino, H.; Kato, M.; Nishiyama, K.; Okada, I.; Yoneda, Y.; Tsurusaki, Y.; Doi, H.; Miyake, N.; Kubota, M.; et al. Paternal mosaicism of an STXBP1 mutation in OS. *Clin. Genet.* **2011**, *80*, 484–488. https://doi.org/10.1111/J.1399-0004.2010.01575.X.
62. Ortega-Moreno, L.; Giráldez, B.G.; Verdú, A.; García-Campos, O.; Sánchez-Martín, G.; Serratosa, J.M.; Guerrero-López, R. Novel mutation in STXBP1 gene in a patient with non-lesional Ohtahara syndrome. *Neurologia* **2015**, *31*, 523–527. https://doi.org/10.1016/j.nrl.2014.10.017.
63. Yamamoto, T.; Shimojima, K.; Yano, T.; Ueda, Y.; Takayama, R.; Ikeda, H.; Imai, K. Loss-of-function mutations of STXBP1 in patients with epileptic encephalopathy. *Brain Dev.* **2016**, *38*, 280–284. https://doi.org/10.1016/j.braindev.2015.09.004.
64. Cogliati, F.; Giorgini, V.; Masciadri, M.; Bonati, M.T.; Marchi, M.; Cracco, I.; Gentilini, D.; Peron, A.; Savini, M.N.; Spaccini, L.; et al. Pathogenic Variants in STXBP1 and in Genes for GABAa Receptor Subunities Cause Atypical Rett/Rett-like Phenotypes. *Int. J. Mol. Sci.* **2019**, *20*, 3621. https://doi.org/10.3390/IJMS20153621.
65. Lykke-Andersen, S.; Jensen, T.H. Nonsense-mediated mRNA decay: an intricate machinery that shapes transcriptomes. *Nat. Rev. Mol. Cell Biol.* **2015**, *16*, 665–677. https://doi.org/10.1038/NRM4063.

66. Misura, K.M.; Scheller, R.H.; Weis, W.I. Three-dimensional structure of the neuronal-Sec1-syntaxin 1a complex. *Nature* **2000**, *404*, 355–362. https://doi.org/10.1038/35006120.
67. Aiken, J.; Buscaglia, G.; Bates, E.A.; Moore, J.K. The α-Tubulin gene TUBA1A in Brain Development: A Key Ingredient in the Neuronal Isotype Blend. *J. Dev. Biol.* **2017**, *5*, 8. https://doi.org/10.3390/JDB5030008.
68. Keays, D.A.; Tian, G.; Poirier, K.; Huang, G.J.; Siebold, C.; Cleak, J.; Oliver, P.L.; Fray, M.; Harvey, R.J.; Molnár, Z.; et al. Mutations in α-Tubulin Cause Abnormal Neuronal Migration in Mice and Lissencephaly in Humans. *Cell* **2007**, *128*, 45–57. https://doi.org/10.1016/j.cell.2006.12.017.
69. Poirier, K.; Keays, D.A.; Francis, F.; Saillour, Y.; Bahi, N.; Manouvrier, S.; Fallet-Bianco, C.; Pasquier, L.; Toutain, A.; Tuy, F.P.D.; et al. Large spectrum of lissencephaly and pachygyria phenotypes resulting from de novo missense mutations in tubulin alpha 1A (TUBA1A). *Hum. Mutat.* **2007**, *28*, 1055–1064. https://doi.org/10.1002/humu.20572.
70. Kumar, R.A.; Pilz, D.T.; Babatz, T.D.; Cushion, T.D.; Harvey, K.; Topf, M.; Yates, L.; Robb, S.; Uyanik, G.; Mancini, G.M.S.; et al. TUBA1A mutations cause wide spectrum lissencephaly (smooth brain) and suggest that multiple neuronal migration pathways converge on alpha tubulins. *Hum. Mol. Genet.* **2010**, *19*, 2817–2827. https://doi.org/10.1093/hmg/ddq182.
71. Hebebrand, M.; Hüffmeier, U.; Trollmann, R.; Hehr, U.; Uebe, S.; Ekici, A.B.; Kraus, C.; Krumbiegel, M.; Reis, A.; Thiel, C.T.; et al. The mutational and phenotypic spectrum of TUBA1A-associated tubulinopathy. *Orphanet J. Rare Dis.* **2019**, *14*, 38. https://doi.org/10.1186/S13023-019-1020-X.
72. Aiken, J.; Buscaglia, G.; Aiken, A.S.; Moore, J.K.; Bates, E.A. Tubulin mutations in brain development disorders: Why haploinsufficiency does not explain TUBA1A tubulinopathies. *Cytoskeleton* **2020**, *77*, 40–54. https://doi.org/10.1002/CM.21567.
73. Catterall, W.A. From ionic currents to molecular mechanisms: the structure and function of voltage-gated sodium channels. *Neuron* **2000**, *26*, 13–25. https://doi.org/10.1016/S0896-6273(00)81133-2.
74. Mulley, J.C.; Scheffer, I.E.; Petrou, S.; Dibbens, L.M.; Berkovic, S.F.; Harkin, L.A. SCN1A mutations and epilepsy. *Hum. Mutat.* **2005**, *25*, 535–542. https://doi.org/10.1002/humu.20178.
75. Claes, L.R.F.; Deprez, L.; Suls, A.; Baets, J.; Smets, K.; Van Dyck, T.; Deconinck, T.; Jordanova, A.; De Jonghe, P. The SCN1A variant database: A novel research and diagnostic tool. *Hum. Mutat.* **2009**, *30-10*, E904-E920. https://doi.org/10.1002/humu.21083.
76. Meng, H.; Xu, H.Q.; Yu, L.; Lin, G.W.; He, N.; Su, T.; Shi, Y.W.; Li, B.; Wang, J.; Liu, X.R.; et al. The SCN1A Mutation Database: Updating Information and Analysis of the Relationships among Genotype, Functional Alteration, and Phenotype. *Hum. Mutat.* **2015**, *36*, 573–580. https://doi.org/10.1002/humu.22782.
77. Escayg, A.; MacDonald, B.T.; Meisler, M.H.; Baulac, S.; Huberfeld, G.; An-Gourfinkel, I.; Brice, A.; LeGuern, E.; Moulard, B.; Chaigne, D.; et al. Mutations of SCN1A, encoding a neuronal sodium channel, in two families with GEFS+2. *Nat. Genet.* **2000**, *24*, 343–345. https://doi.org/10.1038/74159.
78. Claes, L.; Del-Favero, J.; Ceulemans, B.; Lagae, L.; Van Broeckhoven, C.; De Jonghe, P. De Novo Mutations in the Sodium-Channel Gene SCN1A Cause Severe Myoclonic Epilepsy of Infancy. *Am. J. Hum. Genet.* **2001**, *68*, 1327–1332. https://doi.org/10.1086/320609.
79. Kanai, K.; Hirose; Oguni; Fukuma; Shirasaka; Miyajima; Wada; Iwasa; Yasumoto; Matsuo; et al. Effect of localization of missense mutations in SCN1A on epilepsy phenotype severity. *Neurology* **2004**, *63*, 329–334. https://doi.org/10.1212/01.wnl.0000129829.31179.5b.
80. Ceulemans, B.P.G.M.; Claes, L.R.F.; Lagae, L.G. Clinical correlations of mutations in the SCN1A gene: From febrile seizures to severe myoclonic epilepsy in infancy. *Pediatr. Neurol.* **2004**, *30*, 236–243. https://doi.org/10.1016/j.pediatrneurol.2003.10.012.
81. de Lange, I.M.; Koudijs, M.J.; van 't Slot, R.; Gunning, B.; Sonsma, A.C.M.; van Gemert, L.J.J.M.; Mulder, F.; Carbo, E.C.; van Kempen, M.J.A.; Verbeek, N.E.; et al. Mosaicism of de novo pathogenic SCN1A variants in epilepsy is a frequent phenomenon that correlates with variable phenotypes. *Epilepsia* **2018**, *59*, 690–703. https://doi.org/10.1111/EPI.14021.
82. Escayg, A.; Goldin, A.L. Sodium channel SCN1A and epilepsy: Mutations and mechanisms. *Epilepsia* **2010**, *51*, 1650–1658. https://doi.org/10.1111/j.1528-1167.2010.02640.x.
83. Ohmori, I.; Ouchida, M.; Ohtsuka, Y.; Oka, E.; Shimizu, K. Significant correlation of the SCN1A mutations and severe myoclonic epilepsy in infancy. *Biochem. Biophys. Res. Commun.* **2002**, *295*, 17–23. https://doi.org/10.1016/S0006-291X(02)00617-4.
84. Gurrieri, F.; Franco, B.; Toriello, H.; Neri, G. Oral-facial-digital syndromes: review and diagnostic guidelines. *Am J Med Genet A* **2007**, *143A*, 3314–3323. https://doi.org/10.1002/ajmg.a.32032.
85. Franco, B.; Thauvin-Robinet, C. Update on oral-facial-digital syndromes (OFDS). *Cilia* **2016**, *5*, 12. https://doi.org/10.1186/s13630-016-0034-4.
86. Bruel, A.L.; Franco, B.; Duffourd, Y.; Thevenon, J.; Jego, L.; Lopez, E.; Deleuze, J.F.; Doummar, D.; Giles, R.H.; Johnson, C.A.; et al. Fifteen years of research on oral-facial-digital syndromes: from 1 to 16 causal genes. *J. Med. Genet.* **2017**, *54*, 371–380. https://doi.org/10.1136/jmedgenet-2016-104436.
87. Strong, A.; Simone, L.; Krentz, A.; Vaccaro, C.; Watson, D.; Ron, H.; Kalish, J.M.; Pedro, H.F.; Zackai, E.H.; Hakonarson, H. Expanding the genetic landscape of oral-facial-digital syndrome with two novel genes. *Am. J. Med. Genet. A* **2021**, *185*, 2409–2416. https://doi.org/10.1002/ajmg.a.62337.
88. Lee, H.; Deignan, J.L.; Dorrani, N.; Strom, S.P.; Kantarci, S.; Quintero-Rivera, F.; Das, K.; Toy, T.; Harry, B.; Yourshaw, M.; et al. Clinical exome sequencing for genetic identification of rare Mendelian disorders. *JAMA* **2014**, *312*, 1880–1887. https://doi.org/10.1001/JAMA.2014.14604.

89. Wright, C.F.; Fitzgerald, T.W.; Jones, W.D.; Clayton, S.; McRae, J.F.; Van Kogelenberg, M.; King, D.A.; Ambridge, K.; Barrett, D.M.; Bayzetinova, T.; et al. Genetic diagnosis of developmental disorders in the DDD study: a scalable analysis of genome-wide research data. *Lancet* **2015**, *385*, 1305–1305. https://doi.org/10.1016/S0140-6736(14)61705-0.
90. Burdick, K.J.; Cogan, J.D.; Rives, L.C.; Robertson, A.K.; Koziura, M.E.; Brokamp, E.; Duncan, L.; Hannig, V.; Pfotenhauer, J.; Vanzo, R.; et al. Limitations of exome sequencing in detecting rare and undiagnosed diseases. *Am. J. Med. Genet. A.* **2020**, *182*, 1400–1406. https://doi.org/10.1002/ajmg.a.61558.
91. Belkadi, A.; Bolze, A.; Itan, Y.; Cobat, A.; Vincent, Q.B.; Antipenko, A.; Shang, L.; Boisson, B.; Casanova, J.L.; Abel, L. Whole-genome sequencing is more powerful than whole-exome sequencing for detecting exome variants. *Proc. Natl. Acad. Sci. USA* **2015**, *112*, 5473–5478. https://doi.org/10.1073/pnas.1418631112.
92. Meienberg, J.; Bruggmann, R.; Oexle, K.; Matyas, G. Clinical sequencing: is WGS the better WES? *Hum. Genet.* **2016**, *135*, 359–362. https://doi.org/10.1007/S00439-015-1631-9.
93. Ebbert, M.T.W.; Jensen, T.D.; Jansen-West, K.; Sens, J.P.; Reddy, J.S.; Ridge, P.G.; Kauwe, J.S.K.; Belzil, V.; Pregent, L.; Carrasquillo, M.M.; et al. Systematic analysis of dark and camouflaged genes reveals disease-relevant genes hiding in plain sight. *Genome Biol.* **2019**, *20*, 97–97. https://doi.org/10.1186/s13059-019-1707-2.
94. Mantere, T.; Kersten, S.; Hoischen, A. Long-Read Sequencing Emerging in Medical Genetics. *Front. Genet.* **2019**, *10*, 426. https://doi.org/10.3389/fgene.2019.00426.
95. Miller, D.E.; Sulovari, A.; Wang, T.; Loucks, H.; Hoekzema, K.; Munson, K.M.; Lewis, A.P.; Fuerte, E.P.A.; Paschal, C.R.; Walsh, T.; et al. Targeted long-read sequencing identifies missing disease-causing variation. *Am. J. Hum. Genet.* **2021**, *108*, 1436–1449. https://doi.org/10.1016/j.ajhg.2021.06.006.
96. Mantere, T.; Neveling, K.; Pebrel-Richard, C.; Benoist, M.; van der Zande, G.; Kater-Baats, E.; Baatout, I.; van Beek, R.; Yammine, T.; Oorsprong, M.; et al. Optical genome mapping enables constitutional chromosomal aberration detection. *Am. J. Hum. Genet.* **2021**, *108*, 1409–1422. https://doi.org/10.1016/j.ajhg.2021.05.012.

Disclaimer/Publisher's Note: The statements, opinions and data contained in all publications are solely those of the individual author(s) and contributor(s) and not of MDPI and/or the editor(s). MDPI and/or the editor(s) disclaim responsibility for any injury to people or property resulting from any ideas, methods, instructions or products referred to in the content.
